# Routes of postoperative analgesia administration in surgical wards: practice vs. guidelines and economic implications

**DOI:** 10.3389/fmed.2025.1660365

**Published:** 2025-10-31

**Authors:** Suzana Bojic, Nebojsa Ladjevic, Ivan Palibrk, Nemanja Rancic, Mihailo Bezmarevic, Winfried Meissner, Ruth Zaslansky, Ulrike M. Stamer, Philipp Baumbach, Dusica Stamenkovic

**Affiliations:** ^1^Department of Anesthesiology and Intensive Care, University Hospital Medical Centre “Dr. Dragisa Misovic – Dedinje”, Belgrade, Serbia; ^2^Faculty of Medicine, University of Belgrade, Belgrade, Serbia; ^3^Department of Anesthesiology and Intensive Care, University Clinical Centre of Serbia, Belgrade, Serbia; ^4^Medical Faculty of Military Medical Academy, University of Defense, Belgrade, Serbia; ^5^Centre for Clinical Pharmacology, Military Medical Academy, Belgrade, Serbia; ^6^Department of Hepatobiliary and Pancreatic Surgery, Unit for Perioperative Nutrition, Clinic for General Surgery, Military Medical Academy, University of Defense, Belgrade, Serbia; ^7^Department of Anesthesiology and Intensive Care, University Hospital Jena, Jena, Germany; ^8^Department of Anesthesiology and Pain Medicine, Inselspital, Bern University Hospital, Bern, Switzerland; ^9^Department of Biomedical Research, University of Bern, Bern, Switzerland; ^10^Department of Anesthesiology and Intensive Care, Military Medical Academy, Belgrade, Serbia

**Keywords:** analgesia, cost, intravenous, oral route, postoperative, route, surgery

## Abstract

**Introduction:**

Multimodal analgesia, critical to postoperative recovery, typically involves oral (PO), intravenous (IV), or regional analgesia. The PO route is preferred for its non-invasive nature, cost-effectiveness, and comparable efficacy to parenteral routes. This study assessed whether analgesic practices in surgical wards align with current guidelines and evaluated the economic impact of deviations.

**Materials and methods:**

Data from 2,354 adult patients, collected using the PAIN OUT methodology across 10 Serbian hospitals, were analyzed. Patients undergoing various surgeries were observed during the first 24 h postoperatively. We analyzed analgesic administration routes on surgical wards for the entire cohort and a subgroup where PO administration was deemed feasible. Potential savings of transitioning from parenteral to PO were calculated, including medication, disposables, and labor costs.

**Results:**

In the entire cohort, the IV route was used in 86% of patients receiving non-opioids and 39% receiving opioids, while the IM route was used in 7 and 4%, respectively. The PO route was employed in only 1% of patients. Among 1,850 patients for whom the PO route was considered feasible, the IV route was used in 86% for non-opioids and 38% for opioids, and the IM route in 8 and 5%, respectively. Transitioning from parenteral to PO administration would reduce costs from 822.3 RSD (7.84 USD) to 124.5 RSD (1.19 USD) per patient, resulting in 85% savings.

**Conclusion:**

The reliance on IV analgesia and underuse of PO route in Serbia reveals a gap between practice and guidelines. Transitioning to PO analgesia could result in significant cost savings.

## 1 Introduction

Effective acute postoperative pain management is paramount for ensuring patient comfort, expediting recovery, and minimizing complications (1). Central to this objective is the adoption of multimodal analgesia, which integrates diverse medications and techniques for pain relief (2). This often includes the administration of multiple analgesics through various routes. The choice of route is influenced by multiple factors, including the type of surgical procedure, individual patient risk profiles, patient education, and hospital resources such as availability of medications and delivery technologies, pain management protocols, and staff training (3). The financial implications of route selection may be significant particularly in multimodal analgesia, i.e., combination of opioids and non-opioids (4).

The current approach to postoperative pain management emphasizes the use of regional analgesia (RA) techniques alongside systemic analgesia administered orally (PO) or intravenously (IV) (1, 5). Although RA techniques are relatively straightforward to perform in operating theaters and recovery rooms, their use in surgical wards may be constrained by staff expertise and financial limitations, particularly in low- and middle-income countries (6, 7). Consequently, the primary routes for administering analgesia in surgical wards are typically PO or parenteral. The PO route is favored over IV as soon as patients can resume oral intake (8). This preference is grounded in the convenience, non-invasiveness, and cost-effectiveness of the PO route, while still ensuring comparable efficacy (9). Furthermore, immediate-release opioids are preferred over modified-release formulations due to easier titration (10). The intramuscular (IM) route is discouraged because it causes discomfort and has unreliable absorption, resulting in inconsistent postoperative analgesia (3).

While the recommendations for postoperative analgesic administration routes are well-established, data on adherence to these guidelines in surgical wards are limited. The PAIN OUT registry presents a valuable resource, encompassing data from over 10,000 patients across 10 countries (11), offering an opportunity to evaluate international adherence to these guidelines. In this pilot study, we utilized the Serbian PAIN OUT database to pursue two primary objectives: (1) assess the proportion of patients in surgical wards who received analgesics via PO, IV, or IM routes, or through RA techniques; and (2) estimate the potential savings if the PO route were used instead of parenteral routes in patients for whom oral intake was deemed feasible based on the surgical procedure performed.

## 2 Materials and methods

### 2.1 Study design and settings

We conducted a secondary analysis of data obtained from 2,354 patients during the first 24 h after surgery, who underwent various surgical procedures. The data were collected between January 2018 and December 2019 from 10 government hospitals in Serbia, following the PAIN OUT methodology (12).

Patients were enrolled in PAIN OUT if they met the following inclusion criteria: (1) they were 18 years or older; (2) they were on their first postoperative day and had been back in the ward from the recovery room for at least six hours; and (3) they provided written consent to participate.

For this analysis, we used the following variables from the PAIN OUT registry: the type of surgical procedure, the route of analgesic administration after the patients returned to the ward, and the total dose of opioid and non-opioid analgesics administered during the patients’ first 24 h on the surgical ward after discharge from the recovery room. The term “non-opioids” referred specifically to non-opioid analgesics, including paracetamol, metamizole, nonsteroidal anti-inflammatory drugs (NSAIDs), and cyclooxygenase-2 inhibitors, while excluding co-analgesics such as ketamine, dexamethasone, and dexmedetomidine.

### 2.2 Assessment of analgesic administration routes in surgical wards

As the initial step of the analysis, we calculated the proportion of patients receiving non-opioid and opioid analgesics through regional techniques and via PO, IV, and IM routes in the entire cohort of 2,354 patients.

As a second step, we analyzed the proportion of patients receiving non-opioid and opioid analgesics via IV or IM routes in a subgroup of patients in whom administration of analgesics via PO route was deemed feasible based on a surgical procedure they underwent. The PO route was not considered feasible in patients undergoing gastrointestinal, pancreatic and hepatobiliary surgery except for laparoscopic cholecystectomy.

### 2.3 Calculating costs associated with parenteral administration of analgesics

The estimated costs associated with parenterally administered analgesics included the costs of the parenteral formulations of the medications, the cost of staff labor for preparing and administering the analgesics, and the costs of related disposable materials, according to the following equation:


(1) $$C_{\mathrm{par}}=C_{\mathrm{med}}+C_{\mathrm{lab}}+C_{\mathrm{disp}}$$

where C_par_ represents the total cost of parenteral analgesic administration, C_med_ is the cost of the parenteral medication formulation, C_lab_ is the cost of staff labor, and C_disp_ denotes the cost of disposable materials (e.g., syringes, needles, infusion sets) (4).

The costs of the parenteral formulations of analgesics were determined by multiplying the number of used vials of each medication by the unitary price of each vial. The number of used vials was derived from the cumulative dose of each medication, as recorded per the PAIN OUT protocol, divided by the dose of that medication per vial. If the medication was available in vials with varying doses, the price of the vial containing the larger dose was utilized. In cases where the medication was administered in a dose smaller than that provided by a vial, the cost for the entire vial was considered (4). The cost of staff labor for preparing and administering analgesics was calculated by multiplying the number of vials used by the labor cost for administering medications via IV or IM routes (4).

### 2.4 Calculating the projected costs associated with oral administration of analgesics

We calculated the projected costs of administering analgesics via the oral (PO) route, ensuring the doses were equipotent to those given parenterally. This calculation was based on the IV and IM doses obtained in the previous step of the analysis (9, 13). Since pethidine is not available in PO form, costs related to its administration were calculated using the equianalgesic dose of oral morphine (see Supplementary Table S1). The projected costs for PO analgesic therapy included only the cost of the immediate-release PO formulations (14), as the labor cost for oral administration is 0 RSD and no additional disposables are used, since patients bring their own reusable plastic cups. The projected savings were calculated by comparing the costs of parenterally and orally administered analgesic therapy.

All prices were obtained from the official 2023 price list of the Republic of Serbia National Health Insurance Fund, which defines the reimbursement rates for medications, labor, and disposable materials uniformly across all government hospitals in Serbia. Hospitalization costs and staff fees were considered fixed. We estimated the costs of disposable materials related to analgesic therapy assuming “ideal consumption,” meaning that disposable materials were utilized without any waste.

Costs are expressed in Republic of Serbia Dinars (RSD) and United States Dollars (USD) based on the official conversion rate of the National Bank of Serbia as of December 31st, 2023.

## 3 Results

### 3.1 Assessment of analgesic administration routes in surgical wards

In the entire cohort of 2,354 patients, the IV route was used for administering non-opioids in 2,034 (86%) patients and opioids in 928 (39%) patients. Opioids were also administered as IV Patient-Controlled Analgesia (IV-PCA) in 21 (1%) patients. The IM route was employed for administering non-opioids and opioids in 158 (7%) and 91 (4%) patients, respectively. The PO route was only utilized in 18 (1%) patients, all for immediate-release non-opioids. The RA was limited to epidurals and used in 21 (1%) patients.

### 3.2 Analgesia administration routes in patients in whom PO route was deemed feasible

In a subgroup of 1,850 patients for whom the PO route was considered feasible, the IV route was used in 1,595 patients (86%) for administering non-opioids and in 698 patients (38%) for administering opioids. In the same subgroup, the IM route was used in 148 patients (8%) for non-opioid administration and in 87 patients (5%) for opioid administration.

### 3.3 Projected savings in patients with feasible PO intake

Table 1 presents the total number of IV and IM injections of analgesics administered in surgical wards within the first 24 h after surgery in patients in whom PO administration of analgesics was deemed feasible.

**Table 1:** Parenterally administered analgesics in patients with feasible PO route.

	Intramuscular		Intravenous	
	Number of patients	Number of injections	Number of patients	Number of injections
Non-opioids				
Ketorolac	1 (0.1%)	2	435 (23.5%)	789
Paracetamol	NA	NA	579 (30.9%)	670
Metamizole	9 (0.5%)	24	677 (36.6%)	399
Diclofenac	124 (6.7%)	162	251 (13.6%)	342
Ketoprofen	11 (0.6%)	18	93 (5.0%)	130
Opioids				
Tramadol	58 (3.1%)	83	558 (30.2%)	1,369
Morphine	12 (0.6%)	12	141 (7.6%)	142
Pethidine	17 (0.9%)	26	11 (0.6%)	13

NA – not applicable.

The comparison of costs between the surgical disciplines for parenteral and PO administration of analgesics in equipotent doses is presented in Table 2. The cost of analgesics administered via IV and IM routes averaged at 822.3 RSD (7.84 USD) per patient. In contrast, the projected costs associated with the equianalgesic doses administered via PO route were notably lower, averaging 124.5 RSD (1.19 USD) per patient. On average, the projected reduction in costs is estimated at 85%.

**Table 2:** Costs and projected cost reduction in surgical patients with feasible PO route.

Surgical discipline	Number of patients	Cost of IV and IM analgesics		Projected cost of PO analgesics		Projected cost reduction (%)
		RSD	USD	RSD	USD	
Cardiac	228	192,877.2	1,839.55	24,970.5	238.15	87.1
General	603	345,424.8	3,294.47	78,521.7	748.90	77.3
Obstetrics and gynecology	344	171,425.2	1,634.96	49,981.1	476.69	70.8
Traumatology and orthopedics	475	467,495.3	4,458.71	50,952.6	485.96	89.1
Urology	202	343,975.8	3,280.65	25,819.9	246.26	92.5
All	1850	1,521,198.3	14,508.33	230,245.8	2195.96	84.9

RSD – Republic of Serbia Dinar; USD – United States Dollar.

## 4 Discussion

The present study provides the initial insight into the current landscape of postoperative analgesic administration routes in surgical wards during the initial 24 h after surgery in Serbia. It underscores two key findings: Firstly, clinical practice often diverges from established guidelines by prioritizing parenteral over oral routes of administration, particularly in patients in whom oral intake is deemed feasible based on the surgical procedure they underwent. Secondly, this misalignment carries substantial financial implications, as analgesic administration via the PO route instead of IV or IM could yield savings of up to 85%.

### 4.1 Analgesic administration routes in surgical wards

The overwhelming use of the IV route for administering analgesics in surgical wards within the first 24 h after surgery stands in stark contrast to early recovery after surgery strategies (15, 16). In most surgical disciplines, the decision to initiate oral intake is straightforward because anesthesia and surgical procedures that do not interfere with the gastrointestinal (GI) tract typically do not disrupt the absorption of the orally administered medications (17, 18). Conversely, upper GI surgery (19) and other major GI resections (20, 21) are expected to alter the absorption of analgesics. We took a conservative approach by considering all patients undergoing gastrointestinal, pancreatic, and hepatobiliary surgeries, except for laparoscopic cholecystectomy, as unsuitable for oral analgesics within the first 24 h after surgery. This decision was based on variability in clinical practices, as studies in the scientific literature highlight differing protocols for initiating oral intake across various surgical disciplines (22, 23).

An additional concern that may prevent the use of the PO route in surgical wards is postoperative nausea and vomiting (PONV). We operated under the assumption that this issue was resolved before discharge from the recovery room (24), as the PAIN OUT database records nausea data for the entire 24-h postoperative period, rather than specifically for the time spent in the surgical wards during that period. However, there may have been individual patients on the ward who were also experiencing PONV.

As one of the limitations of our analysis is the lack of data on antiemetics type and dosage in the PAIN OUT database; therefore, we could not analyze the incidence or management of nausea and vomiting. We acknowledge the lower emetogenic potential of slow-release opioid formulations; however, we based our calculations on immediate-release formulations since the pharmacokinetic profile of parenteral administration corresponds more closely to that of immediate-release opioids.

As evident from our data, patients in our cohort typically received multimodal analgesia, most often a combination of opioid and non-opioid agents (4). However, the focus of the present study was specifically on the costs associated with the route of administration of analgesics, particularly the potential savings achievable by shifting from parenteral to oral routes, when feasible. Analyses from the same cohort have already explored both the efficacy and the cost-effectiveness of these multimodal strategies in greater detail, as referenced in our previous publications (4). Of note is that in postoperative analgesia implementation, different analgesic drugs and administration methods exhibit varying efficacy. When the analgesic effects are inconsistent, evaluating the cost of each method may lead to biased results.

The use of PCA and regional blocks for postoperative pain management in presented cohort is sporadic (in less than 1% of patients). This underutilization highlights a major gap in our practice. One of the aims of this study was to provide economic data that could help redirect available resources, such as the savings from reduced parenteral use, toward increasing access to regional and local analgesia methods. One of the broader aims of our research group is to generate reliable cost data, such as those presented in this study, to support dialogue with hospital administrations and policymakers. Demonstrating potential savings from reduced parenteral use could help redirect resources toward acquiring PCA pumps and improving access to advanced analgesia modalities. PCA and regional anesthesia techniques are more commonly employed in high-income countries (25, 26), indicating that their underutilization in our setting may be due to resource limitations, staff education and a need for enhanced training among healthcare providers. Expanding this pilot study to the entire PAIN OUT network database could offer further insights into the differences between high-income and low- to middle-income countries (27). This encompasses not just the availability of medications, but also the utilization and cost-effectiveness of analgesia techniques in surgical wards.

### 4.2 Projected savings in patient with feasible PO intake

The costs of postoperative analgesia may not always compare directly with other surgical expenses. However, the value of cost analysis lies in its capacity to affect patient outcomes and reduce overall healthcare expenditures (4). This study, which evaluated the costs of different routes for administering analgesics in surgical wards within the first 24 h after surgery, indicates that opting for the PO route instead of the parenteral, when possible, could lead to substantial savings, averaging at 85% of analgesia costs. However, it is essential to approach these findings with a nuanced perspective.

The projected cost analysis for oral analgesic administration was based on real-world practices in Serbian hospitals, where staff labor for dispensing oral medications is not reimbursed and patients are required to bring reusable cups. However, this organizational approach is not standard in many healthcare systems and may not be generalizable. In settings where staff time for medication administration is factored into cost calculations, or where single-use disposable cups are used instead of reusable ones, the cost difference between parenteral and oral analgesic therapy would likely be smaller. Furthermore, the routine use of disposable materials for oral administration would also introduce an additional environmental burden, which should be considered when evaluating the broader implications of shifting to PO routes.

Our calculations relied on registry data collected within the first 24 h after surgery. Many surgical patients require a longer hospital stay, leading to extended use of analgesic medications and, consequently, even greater potential savings if the use of parenteral routes continues. As the PAIN OUT protocol does not capture data on complications of parenteral administration (e.g., infection or nerve and vascular injury), our study could not account for the costs associated with their treatment.

Upon analyzing the total number of patients and the administered analgesic doses, it becomes apparent that not every patient received a full daily dosage of non-opioid analgesics. Previous studies have indicated that fewer than half of the Serbian PAIN OUT cohort received a full daily dosage of non-opioid analgesics (12). Utilizing a full daily dosage of non-opioid analgesics as part of perioperative pain management bundle has proven to be cost-effective, improving the overall quality of acute postoperative pain management (4).

Our calculations indicate that substantial cost savings can be achieved for commonly performed surgeries, such as trauma, orthopedic, and urological procedures, if we discontinue administering medications parenterally to patients who are able to take them orally. This aspect has not been previously explored, presenting a novel area for research, especially given the high frequency of these procedures among the aging population.

Transitioning from parenteral to oral systemic analgesia offers the additional advantage of reducing nursing staff workload and increasing patient comfort. Furthermore, it is important to note that a majority of disposables used for parenteral routes of analgesic administration are made of glass or plastic, which has a significant negative environmental impact. For example, recently published data have shown that intravenous paracetamol produces 12-fold higher life-cycle carbon emissions compared to the oral form (28). This is particularly noteworthy since paracetamol was the second most frequently used non-opioid analgesic in our investigation. This dual perspective, although not the primary focus of our study, underscores the importance of considering not only economic benefits but also practical and environmental implications when choosing oral pain medication whenever feasible.

Our findings, based on prices from Serbian government hospitals, may not directly apply to healthcare systems in other countries. Global variations in labor costs, choice and availability of medications, medication prices, and related expenses will impact actual costs and potential savings. However, the model we present for transitioning from parenteral to oral administration of analgesics, along with the associated cost savings, can be applicable across different healthcare systems.

## 5 Conclusion

Our findings reveal a widespread reliance on IV administration for postoperative analgesia on the first day after surgery once patients return to the surgical ward. The limited use of oral analgesics, particularly among eligible patients, highlights a significant gap between current practices and established guidelines. Additionally, the prevalence of the IM route raises concerns. The substantial projected cost savings associated with oral analgesics, estimated at an impressive 85%, underscore the importance of aligning practices with evidence-based recommendations. Expanding the analysis to include multinational data from the PAIN OUT registry could provide further insights into gaps in adherence to available guidelines and lead to actionable measures for improving clinical practices.

## Data Availability

The raw data supporting the conclusions of this article will be made available by the authors, without undue reservation.
