# Supplementary material for: Routes of postoperative analgesia administration in surgical wards: practice vs. guidelines and economic implications
Source: Front Med (Lausanne). 2025 Oct 31;12:1660365. doi: 10.3389/fmed.2025.1660365 (PMC12615410; doi:10.3389/fmed.2025.1660365)
Supplement: Supplementary file 1 [file Table_1.DOCX]

Supplementary Material

**Table S-1. Opioid conversion table**

|  | **Equianalgesic parenteral dose to 10 mg parenteral morphine (mg)** | **Equianalgesic oral dose to 10 mg parenteral morphine (mg)** | **Equianalgesic oral dose to parenteral dose (mg)** |
| --- | --- | --- | --- |
| **Morphine** | 10 | 10 | 10:3.3 |
| **Meperidine** | 75 | 100 | 10:2.5 |
| **Tramadol** | 100 | 3.3 | 1:1 |

Modified from Shaheen PE, Walsh D, Lasheen W, Davis MP, Lagman RL. Opioid equianalgesic tables: are they all equally dangerous? J Pain Symptom Manage. 2009;38(3):409-17. doi: 10.1016/j.jpainsymman.2009.06.004.

**
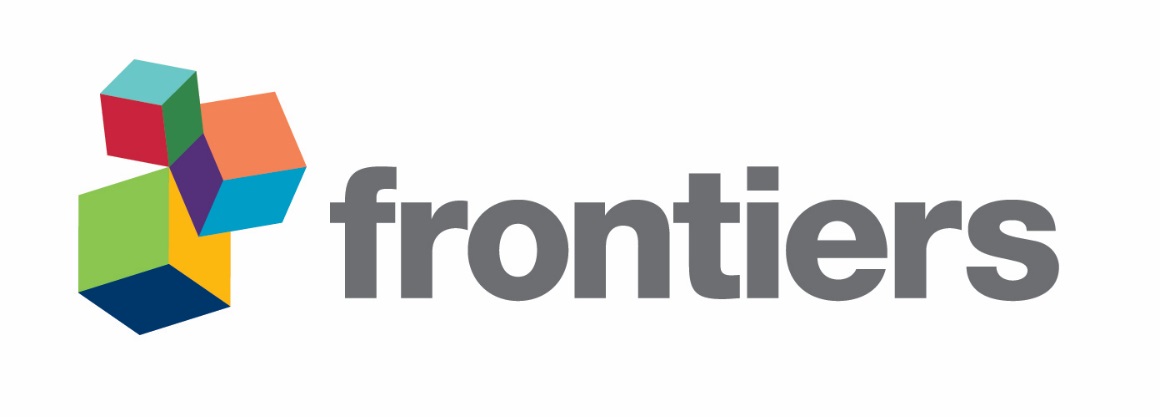
**
